# Severe acidosis due to 5-oxoprolinase inhibition by flucloxacillin in a patient with shoulder prosthesis joint infection

**DOI:** 10.5194/jbji-7-71-2022

**Published:** 2022-04-04

**Authors:** Julia Elisabeth Lenz, Volker Alt, Thomas Dienemann

**Affiliations:** 1 Department of Trauma Surgery, University Medical Center Regensburg, Regensburg, Germany; 2 Department of Surgery, Surgical Intensive Care Unit, University Medical Center Regensburg, Regensburg, Germany

## Abstract

We report a case of a 64-year-old female patient with severe metabolic acidosis. Inhibition of 5-oxoprolinase by flucloxacillin was found to be the cause of the metabolic derailment.

## 1 Introduction

Prosthesis joint infections (PJIs) with multisensitive *Staphylococcus aureus* usually occur as an early infection and are common, accounting for approximately two-thirds of all prosthesis infections (Scheper et al., 2021). In addition to debridement, antibiotic and irrigation (DAIR), two-stage or multiple-stage revision of the endoprosthesis and antibiotic therapy with flucloxacillin or cefazolin – often in combination with rifampicin – for several weeks are the standard therapies for this complication.

A rare potential side effect of therapy with flucloxacillin is metabolic acidosis. We report a case of a 64-year-old female patient with severe metabolic acidosis due to flucloxacillin therapy without comedication with paracetamol.

## 2 Case presentation

A 64-year female patient underwent an aseptic revision of her reverse total shoulder arthroplasty due to notching of the humeral stem into the glenoid. She received a change of the polyethylene inlay and removal of osteophytes. She was previously diagnosed with multiple comorbidities including a chronic pain syndrome, chronic hyponatremia, lupus erythematosus, rheumatoid arthritis, adrenal insufficiency due to long-term cortisone treatment (at the time of reporting, hydrocortisone was 30 mg d ${}^{-1}$ ), a seizure disorder of unclear etiology and Crohn's disease.

Two weeks later she showed symptoms of an acute periprosthetic joint infection at her shoulder (pain, swelling, redness, purulent shoulder punctate, impaired wound healing), for which a debridement, antibiotic and irrigation (DAIR) procedure was performed.

Initially, a calculated i.v. therapy with ampicillin/sulbactam was initiated. A multisensitive *Staphylococcus aureus* was then detected in all tissue samples, and antibiotic therapy was changed to flucloxacillin (6 g d ${}^{-1}$ ) according to its resistogram. The intraoperative histology showed moderate chronic granulation and florid inflammation of the capsular tissue. Rifampicin treatment was not yet indicated as there was still some drainage from the wound.

After 2 weeks of i.v., flucloxacillin therapy, the patient showed subacute tachypnoea, tachycardia and a change in mental status. Upon examination the patient was slender (body mass index 21) with pale skin colour and standing skin folds. Neurologically, the patient was somnolent (Glasgow Coma Scale 10) and dysarthric with tremor at rest. The respiratory rate was 25 min ${}^{-1}$ , and the breathing pattern was deepened (Kussmaul breathing). The patient was tachycardic (heart rate 120 min ${}^{-1}$ ), hypotensive (systolic blood pressure 90 mmHg) and normothermic. The blood gas analysis showed marked metabolic acidosis (see Table 1).

**Table 1:** Venous blood gas analysis and laboratory values on admission of the patient to the intensive care unit. Bold font: laboratory values outside the reference range. GFR represents glomerular filtration rate, CRP represents C-reactive protein, PCT represents procalcitonin, and LDH represents lactate dehydrogenase.

Parameter (unit)	On admission	Reference range
pH	**6.9**	7.35–7.45
p CO ${}_{2}$ (mmHg)	**14.8**	37–50
p O ${}_{2}$ (mmHg)	58.4	36–44
Bicarbonate (mmol L ${}^{-1}$ )	5.5	22–26
Standard base excess (mmol L ${}^{-1}$ )	-26.0	-2 – +3
Anion gap (mmol L ${}^{-1}$ )	**18.3**	8–12
Lactate (mg dL ${}^{-1}$ )	6	5–15
Albumin (g L ${}^{-1}$ )	**26.5**	35–52
Na ${}^{+}$ (mmol L ${}^{-1}$ )	**150**	135–145
K ${}^{+}$ (mmol L ${}^{-1}$ )	3.9	3.5–4.5
Cl ${}^{-}$ (mmol L ${}^{-1}$ )	**128**	98–112
Creatinine (mg dL ${}^{-1}$ )	**1.44**	0.51–0.95
GFR (mL min ${}^{-1}$ m ${}^{-2}$ )	**50**	>85
CRP (mg L ${}^{-1}$ )	**36.8**	<5
Leukocytes (nL ${}^{-1}$ )	**29.48**	3.98–10
PCT (ng mL ${}^{-1}$ )	**0.26**	<0.06
LDH (U L ${}^{-1}$ )	**320**	0–250
Haemoglobin (g dL ${}^{-1}$ )	**6.9**	11.2–15.7
Haptoglobin (mg dL ${}^{-1}$ )	**11.3**	30–200

The patient was admitted to the intensive care unit. With the pre-existing diagnosis of adrenal insufficiency, 100 mg of hydrocortisone was administered in order to rule out development of Addison's crisis. A computer tomography of the skull and thorax excluded an intracranial or pulmonary cause of the dyspnoea and the mental status change. The administration of sodium bicarbonate raised the pH to 7.2 within a few hours, and the compensatory tachypnoea improved.

Differential diagnoses of metabolic acidosis with increased anion gap were examined. All possible causes except oxoprolinaemia were excluded. A laboratory chemical analysis of the urine for 5-oxoproline was ordered for forensic reasons; however, the result notification took 2 weeks due to the time-consuming examination in an external laboratory. During the literature review, we came across a few case reports describing the occurrence of severe acidosis after treatment with flucloxacillin combined with paracetamol. The patient's comedication did not include any drugs with metabolic acidosis listed as adverse events. Furthermore, the medication list did not include paracetamol.

Due to the leucocytosis and the initially unclear clinical picture, sepsis could not be ruled out, so the antibiotic therapy was changed to meropenem and vancomycin.

Unfortunately, the somnolence did not resolve quickly. Most likely due to the altered mental status, the patient suffered an aspiration pneumonia, requiring intubation 4 d after admission to the intensive care unit. Mechanical ventilation was required for 6 d.

Eventually, the acidosis improved under buffering, but the pH did not return to normal values in the first week. Laboratory signs suggestive of haemolysis and leukocytosis disappeared after 2 d. In the absence of clinical signs of an infection, the antibiotic therapy was de-escalated to piperacillin/tazobactam after 1 d. In order to optimise the treatment for the staphylococcal infection, the antibiotic regimen was switched to cefazolin 2 g twice daily and rifampicin 600 mg once daily after having consulted our departments for infectious diseases.

After discharge to the regular ward following 19 d of ICU treatment, we received the external laboratory report. It showed an isolated, massive oxoprolinuria of 2965 mg g ${}^{-1}$ (creatinine) (reference <60 mg g ${}^{-1}$ (creatinine)). The antibiotic treatment was switched to an oral treatment on moxifloxacin 400 mg d ${}^{-1}$ and rifampicin 600 mg d ${}^{-1}$ , and it was given for a further 7 weeks (12 weeks antibiotic treatment in total). The patient showed a complete recovery from the metabolic acidosis and no signs of relapse of the PJI during a hospitalisation for another illness 6 months later.

## 3 Discussion

**Figure 1:**
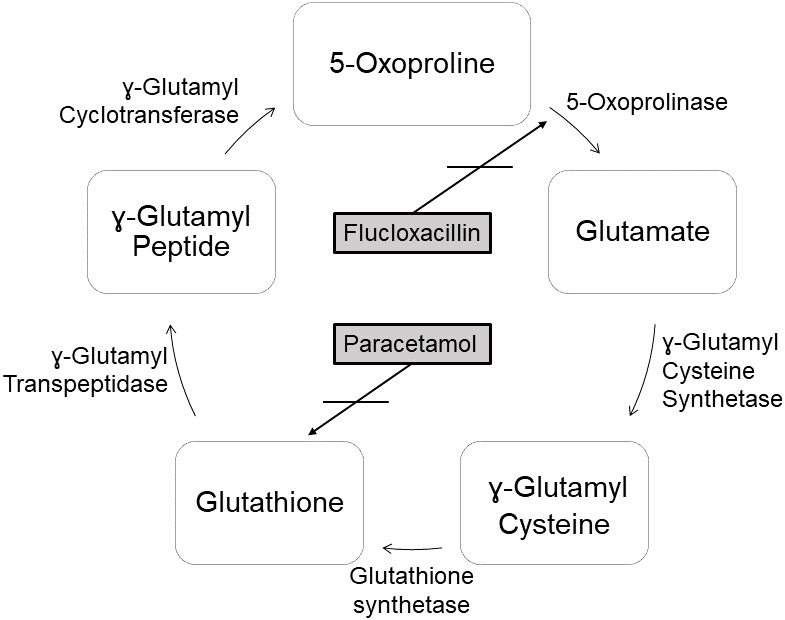
Glutamate–glutathione cycle (Emmett, 2014; Osborne et al., 2019; Weiler et al., 2015).

Metabolic acidosis is a very rare potential side effect of flucloxacillin therapy. This is caused by the accumulation of 5-oxoproline. 5-Oxoproline, also known as pyroglutamic acid, is a derivative of glutamic acid and is formed as an intermediate in glutathione biosynthesis (Emmett, 2014). Glutathione is an important antioxidant that is found in high concentrations in almost all mammalian cells (Emmett, 2014). Normally, 5-oxoproline is degraded to glutamic acid by the enzyme 5-oxoprolinase (see Fig. 1). However, the glutamate–glutathione cycle can be disturbed by various drugs, which leads to an accumulation of 5-oxoproline. In addition to flucloxacillin, paracetamol, ciprofloxacin, the aminoglycoside antibiotic netilmicin and the anticonvulsant vigabatrin are important drugs which could potentially impair this pathway (Schurmans et al., 2014; Veldhuijzen et al., 2012). In the literature, this phenomenon is described particularly in older women on a combination therapy of flucloxacillin and paracetamol. Other influencing factors are liver insufficiency, malnutrition or sepsis (Emmett, 2014; Liss et al., 2013; Jessurun et al., 2016; Berbee et al., 2017). However, metabolic acidosis is currently not yet recorded as a side effect of flucloxacillin by German drug agencies.

Causes of metabolic acidosis with an elevated anion gap can be identified with the help of the GOLDMARK mnemonic, and appropriate therapies can be initiated (Schurmans et al., 2014). In the present case, it was not possible to obtain a medical history of the patient due to the severe somnolence. However, ingestion with glycols, methanol or aspirin during the inpatient stay could be excluded. A normal lactate on blood gas analysis ruled out L-lactatemia as a differential diagnosis. The urine examination ruled out ketonuria. Short bowel syndrome as a risk factor for D-lactatemia did not apply to the patient. With a chronic renal insufficiency (eGFR around 50 mL min ${}^{-1}$ ), a renal cause of anion gap acidosis was unlikely (Weiler et al., 2015). Thus, by exclusion and medication history, the cause of acidosis could be narrowed down to oxoprolinaemia.

Several case reports have shown that metabolic acidosis can develop weeks after the start of flucloxacillin therapy with a range from a few days to 2 months after the start of therapy (Veldhuijzen et al., 2012; Tummers et al., 2020; Hundemer and Fenves, 2017; Luyasu et al., 2014; Agrawal et al., 2017). Acidosis can also persist for several weeks if 5-oxoproline is slowly degraded.

The detection of 5-oxoproline to definitively determine the cause is useful but often not possible by the in-house laboratory as it usually takes several days to weeks, so therapy must already be initiated without reliable evidence of an impairment in glutathione synthesis. In this case, it is important to stop the causing medication immediately. Other penicillin antibiotics such as amoxicillin have not been reported to induce acidosis associated with 5-oxoproline (Zand Irani et al., 2021).

In the case of severe symptoms, intensive care admission for monitoring should be considered. Buffering, e.g. with sodium bicarbonate, should be performed if the symptoms are clinically relevant (Matyukhin et al., 2020; Adeva-Andany et al., 2014). In the case of refractory acidosis, the administration of N-acetylcysteine can be considered (Hundemer and Fenves, 2017; Tummers et al., 2020). Haemodialysis has been shown to accelerate the elimination of 5-oxoproline in case reports (Luyasu et al., 2014; Agrawal et al., 2017).

## 4 Conclusion

To our knowledge, the present case is the first case of life-threatening metabolic acidosis due to 5-oxoproline accumulation from flucloxacillin monotherapy without supplemental paracetamol or other aggravating drugs. In patients on flucloxacillin therapy, metabolic acidosis should always be considered as a differential diagnosis of tachypnoea or decreased vigilance.

## Data Availability

The data that support the findings of this study are available from the corresponding author, Julia Elisabeth Lenz, upon reasonable request.
